# House-wall modification after indoor residual spraying in Shashogo district, southern Ethiopia

**DOI:** 10.1186/s12936-023-04759-0

**Published:** 2023-10-31

**Authors:** Melesech Amanuel, Sebsibe Tadesse, Aiggan Tamene

**Affiliations:** 1 School of Public Health, Wachemo University, Hossana, Ethiopia; 2 National Data Management and Analytics Center for Health, Ethiopian Public Health Institute, Addis Ababa, Ethiopia

**Keywords:** House-wall modification, Indoor residual spraying, Malaria prevention, Ethiopia

## Abstract

**Background:**

Indoor residual spraying has been a key national malaria prevention and control strategy in Ethiopia. However, there is a gap in monitoring and evaluation of house-wall modification after indoor residual spraying before the end of residual lifespan. This study has determined the prevalence of house-wall modification after indoor residual spraying and identified the associated factors in Shashogo district, southern Ethiopia.

**Methods:**

A community-based cross-sectional study was conducted from April to May 2022. Data were collected from 640 randomly selected households using a pre-tested questionnaire and an observational checklist. The binary logistic regression models were used to identify factors associated with house-wall modification after indoor residual spraying before the end of the potency period.

**Results:**

The prevalence of house-wall modification after indoor residual spraying was found to be 30.4% (95% CI 27.4–34.2%). Educational status of could not read and write [AOR = 1.76, 95% CI (1.16, 2.68)], monthly income of more than birr 3000 [AOR = 3.27, 95% CI (1.78, 6.01)], low level of knowledge about indoor residual spraying [AOR = 3.81, 95% CI (2.39, 6.06)], lack of information within two weeks before spraying [AOR = 2.23, 95% CI (1.44, 3.46)], absence of supervision after spraying [AOR = 1.79, 95% CI (1.14, 2.81)], absence of stagnant water near house [AOR = 3.36, 95% CI (2.13, 5.39)], and thatched roof [AOR = 1.82, 95% CI (1.04, 3.16)] were factors significantly associated with house-wall modification after indoor residual spraying.

**Conclusion:**

This study has revealed that the prevalence of house-wall modification after indoor residual spraying before the end of the residual lifespan in the study area was higher compared to other studies in developing countries. Therefore, special emphasis should be given to providing community education about indoor residual spraying, conducting regular supervision before and after residual spraying, enforcing some legislative strategies for modifying the house-wall before six months after spraying, and improving environmental and housing conditions.

## Background

Malaria is the most significant vector-borne disease transmitted to humans through the bite of infected female Anopheles mosquitoes. There were an estimated 241 million cases and 627,000 deaths due to malaria globally in 2020, which represented about 14 million more cases and 69,000 more deaths compared to 2019 [1]. Sub-Saharan Africa accounted for 95% of the cases and 96% of the deaths [1, 2]. Ethiopia shared the highest burden of malaria infections in East Africa. An estimated 75% of the country is malarious, and about 52% of the population is at high risk of malaria infection [1]. *Plasmodium falciparum* and *Plasmodium vivax* are the predominant species. Malaria transmission peaks biannually from September to December and April to June [2].

Malaria has received international attention as a public health priority. It is included under United Nations Sustainable Development Goal Three, which states “End the epidemics of malaria by 2030”. Moreover, the World Health Organization (WHO) has adopted the 2016–2030 global strategy for malaria and recommended the use of indoor residual spraying to control mosquito vectors [3]. Consequently, millions of lives have been saved by implementing the indoor residual spraying programme in Africa, America, Asia, and Europe [4]. Indoor residual spraying has also been a key national malaria prevention and control strategy in Ethiopia [2]. Currently, carbamate insecticides, such as bendiocarb and propoxers, are in use for indoor residual spraying [3].

The training guideline for indoor residual spraying in Ethiopia clearly states that every spraying campaign needs to be followed by an evaluation survey in order to determine the extent of wall modifications by the households and take the appropriate corrective actions. However, such a routine monitoring and evaluation system has not been so far implemented in the country [2, 3].

This study has determined the prevalence of house-wall modification after indoor residual spraying and identified the associated factors in Shashogo district, southern Ethiopia, where malaria was ranked as the second leading cause of morbidity [5].

## Methods

### Study area and period

**Fig. 1:**
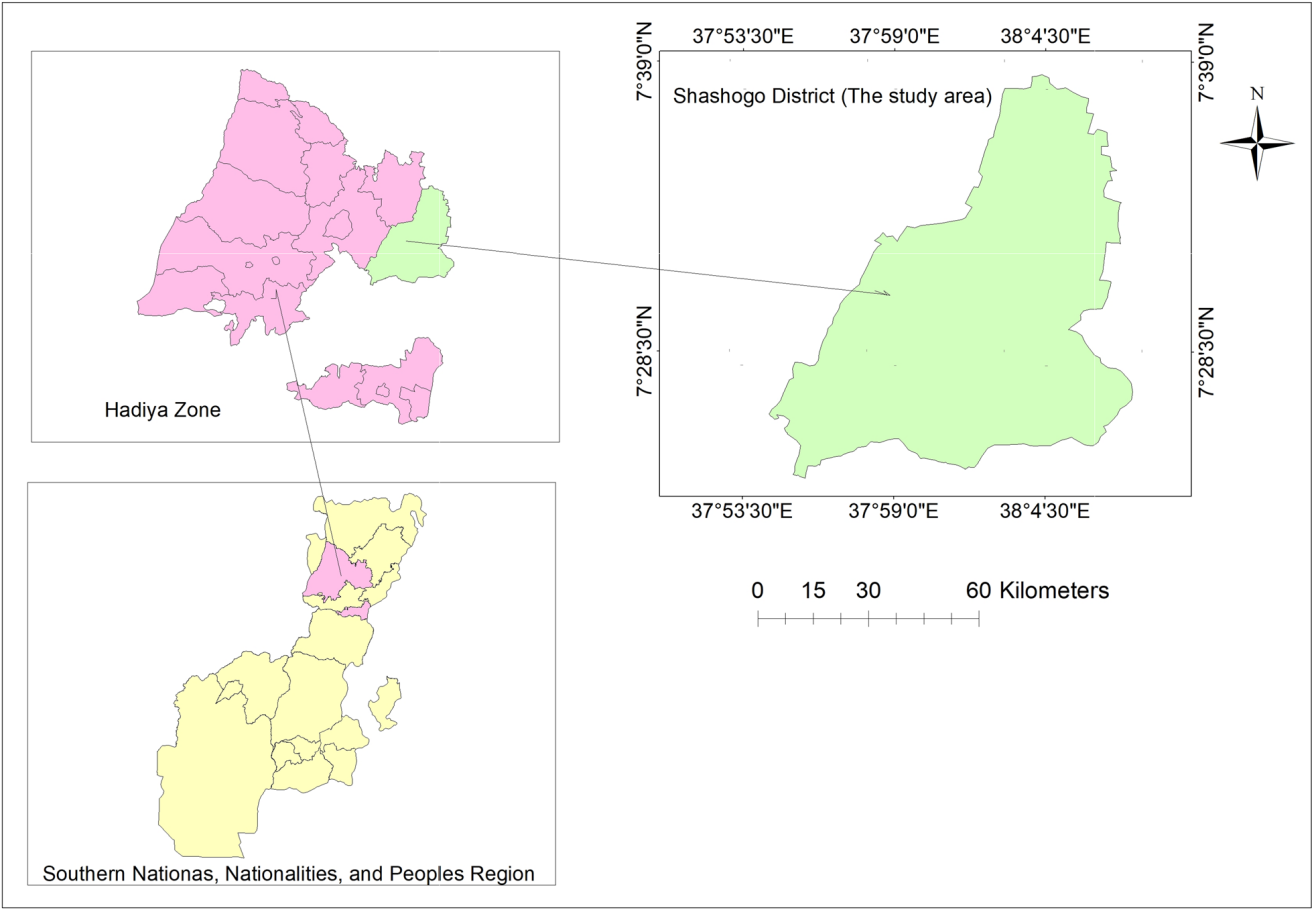
Map of the study area (Shashogo District)

The study was conducted in Shashogo district, southern Ethiopia, from April to May 2022 (Fig. 1). The district had an estimated population of 148,503 people living in 33 rural and three urban villages, or kebeles (the smallest administrative unit with a population of 5000 on average). There are five health centres, one district hospital, and ten private clinics in the study area [5].

### Study design

A population-based cross-sectional study design was used to determine the prevalence of house-wall modification after indoor residual spraying and identify the associated factors in Shashogo district, southern Ethiopia.

### Study population

All households that were found in five selected kebeles for at least six months during and after the indoor residual spraying were included in the study. Households where there were no adult people who could give adequate responses to the interview questions were excluded from the study.

### Sample size determination

Epi Info version 7 was used to determine the minimum sample size of 640 by taking the 50% expected proportion of indoor residual spraying, 5% margin of error, 95% confidence level, 1.5 design effects, and 10% non-response rate.

### Sampling procedure

The multistage sampling technique was used to select the households. Five kebeles were selected by the lottery method from 11 indoor residual sprayed kebeles in the Shashogo district. Then, a total of 640 household samples were proportionally allocated to each selected kebeles (i.e., 125 for Urbacha, 100 for Golicho, 119 for Mololicho, 155 for Suta, and 141 for Bidika). Then, from each kebele, the first household was picked by the lottery method, and the next was drawn with a sixth interval (K = N/n = 3850/640 = 6) in the sampling frame.

### Data collection tools

Data on socio-demographic characteristics, household head’s knowledge about malaria, and environmental and health-related factors were collected using a pre-tested questionnaire. An observational checklist was also used to collect data on the status of wall modification, type of roof, and type of wall surface.

### Data quality control

Data quality was ensured before, during, and after data collection. The training of supervisors and data collectors addressed issues such as field methods, inclusion-exclusion criteria, data collection instruments, and record keeping. The interview questionnaire was pilot-tested on 32 households that had characteristics nearly similar to households in the Shashogo district in order to identify cultural objections and unanticipated interpretations of any of the questions. The questionnaire had a score of 0.81 Cronbach’s alpha. Every day, the collected data were spot-checked by the field supervisors to ensure completeness and consistency.

### Data management and statistical analyses

The data were entered into Epi Info version 3.5.3 statistical software and analysed on SPSS version 20. Tables and graphs were used to present the findings by frequency and percentage. All independent variables were fitted separately into a bivariate logistic model to estimate the degree of association with house-wall modification after indoor residual spraying. Then, variables with a p value < 0.25 were exported to a multivariable logistic model to control for confounders. The statistical significance of the variables was declared by using the odds ratio (OR) with a 95% confidence interval (CI).

### Operational definitions

#### House-wall modification

The act of painting, plastering, or covering the house wall before six months of indoor residual spraying.

#### Indoor residual spraying

The application of insecticides with residual effects for about six months on the walls, roofs, and animal shelters of all houses in a given area in order to kill the mosquitoes that rest on these surfaces [6].

#### Knowledge level of household heads

Household participants who correctly responded to 60% and above on the knowledge assessment questions were categorized as having good knowledge about the importance of indoor residual spraying [7].

## Results

### Socio-demographic characteristics

A total of 621 participants completed the study questionnaire, making the response rate 97%. Of whom, 83.9% were males. The mean age with a standard deviation was 40 ± 9. About 42.6% of the participants belonged to the age group of 36–45 years. The majorities of the participants were married (86.3%), could not read or write (55.6%), and were farmers (86.8%). More than three-fourths (79.2%) had a monthly income of a maximum of birr 3000 (Table 1).

**Table 1:** Socio-demographic characteristics of household heads in Shashogo district, southern Ethiopia, 2022

Variables	Frequency	Percent
Gender		
Male	521	83.9
Female	100	16.1
Age (in years)		
≤ 35	170	27.4
36–45	265	42.6
> 45	186	30.0
Marital status		
Married	578	93.1
Other	43	6.9
Educational status		
Cannot read and write	345	55.6
Can read and write	276	44.4
Occupational status		
Farmer	539	86.8
Other	82	13.2
Monthly income (in Birr)		
≤ 3000	492	79.2
> 3000	129	20.8

### Household heads’ knowledge of malaria

The majority (87.4%) of the participants responded that malaria has been caused by mosquito bites. The common types of malaria preventive measures were insecticide-treated nets (67.5%), indoor residual spraying (52.8%), and drainage of stagnant water (27.7%). About 40.4% of the participants believed that the mosquitoes mostly rested outside the house, followed by 33.5% on walls inside the house and 26.1% in the water body (Table 2).

**Table 2:** Household heads’ knowledge about the cause, preventive measures, and resting place of malaria mosquitoes in Shashogo district, southern Ethiopia, 2022

Variables	Frequency	Percent
Main cause of malaria		
Mosquito bite	543	87.4
Cold	30	4.8
Drainage of stagnant water	24	3.9
Rain	24	3.9
Malaria preventive measures		
Use insecticide treated nets	419	67.5
Use indoor residual spraying	328	52.8
Drainage of stagnant water	172	27.7
Take tablet	54	8.7
Other	80	13.0
Mosquito mostly rest on		
Outside the house	251	40.4
In walls inside the house	208	33.5
In water body	162	26.1

### Household heads’ knowledge about indoor residual spraying

More than three-fourths (77.6%) of the household heads got information about indoor residual spraying from the health extension workers. The majority (92.3%) believed that the indoor residual spraying prevented malaria transmission by killing the mosquitoes. More than two-thirds (72.5%) reported that the spraying was done once a year. About 36.9% believed that the residual would have an effect for at least six months. More than half (55.7%) had a low level of knowledge about the indoor residual effect (Table 3).

**Table 3:** Household heads’ knowledge about indoor residual spraying in Shashogo district, southern Ethiopia, 2022

Variables	Frequency	Percent
Source of information		
Health extension workers	482	77.6
One to five leader	129	21.2
Community event	92	14.8
Other	30	4.8
Indoor residual spraying prevents malaria		
By killing mosquitoes	573	92.3
By other mechanism	48	7.7
Frequency of spraying		
Once in a year	450	72.5
Only once	81	13.0
Every 6 months	48	7.7
Do not know	42	6.8
Duration of the residual effect		
For ≥ 6 months	229	36.9
For 3–5 months	226	36.4
For < 1 month	166	26.7
Level of knowledge		
Low knowledge	346	55.7
High knowledge	275	44.3

### Environmental and health-related conditions

Three hundred seventy-four (60.2%) houses were roofed with grass (thatched roofs), and 39.8% were roofed with corrugated iron sheets. The majority (95.7%) of the houses had rough walls. Of the 67% of houses where mosquito breeding sites existed, 80.6% were found within five minutes of walking distance from the breeding sites. Information prior to the spraying date was given to about 62.5% of the households. Supervision was conducted for more than half (58.3%) of the households after the residual spraying.

### Prevalence of house-wall modification after indoor residual spraying

**Fig. 2:**
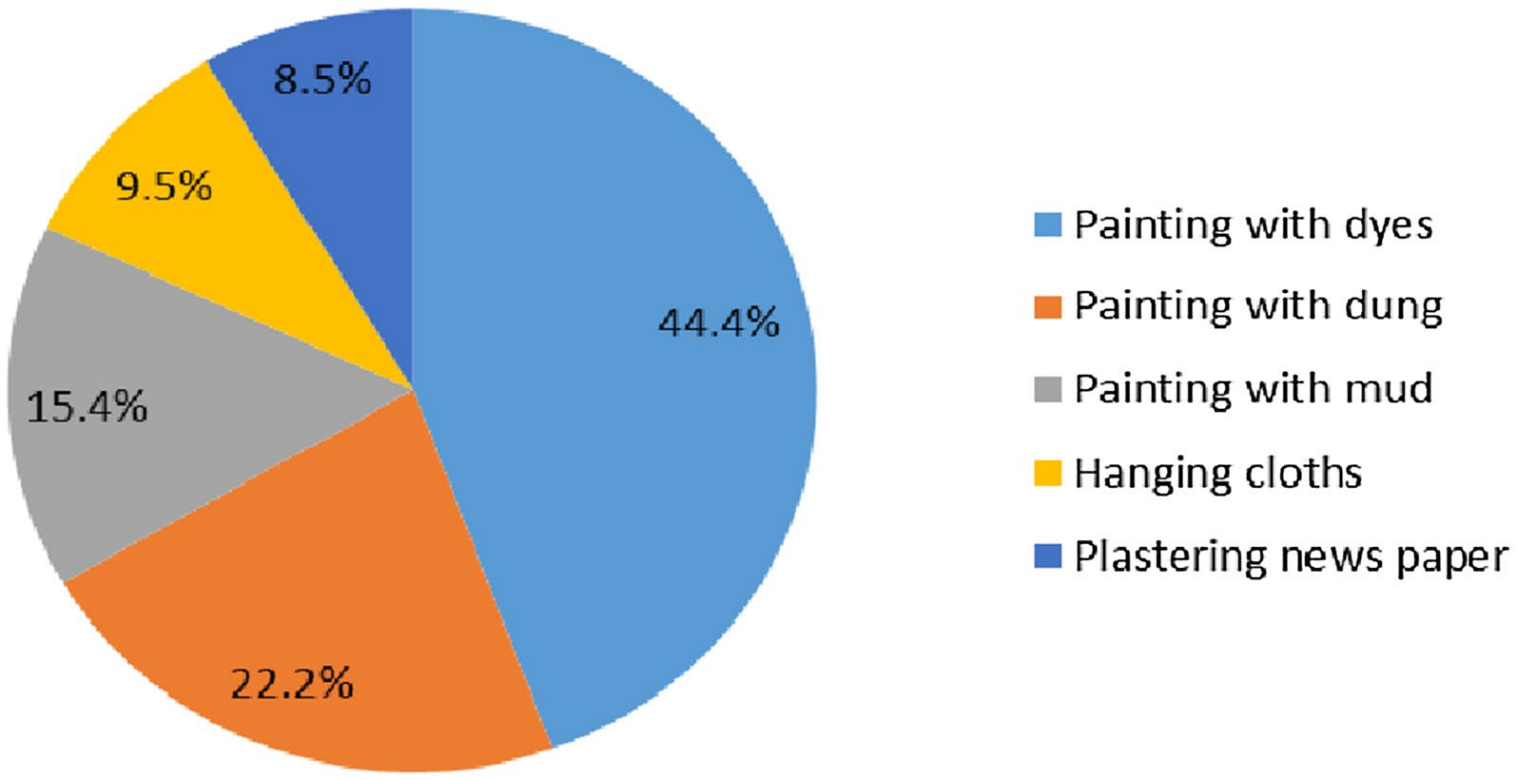
Type of modifications made on house-walls after indoor residual spraying in Shashogo district, southern Ethiopia, 2022

**Fig. 3:**
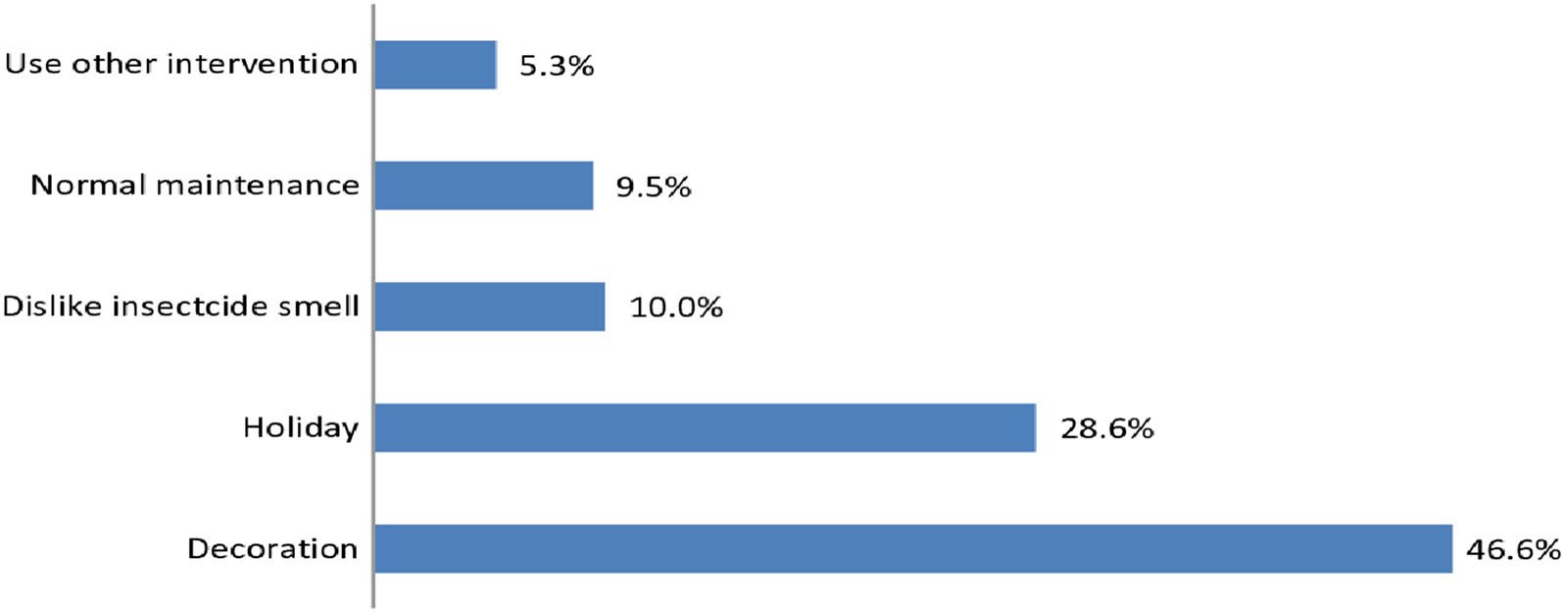
Reasons for house-walls modifications after indoor residual spraying in Shashogo district, southern Ethiopia, 2022

The prevalence of house-wall modification after indoor residual spraying was found to be 30.4% (95% CI 27.4–34.2%). The common types of house-wall modifications before the end of the sprayed chemical’s residual lifespan were painting with dyes (44.4%), painting with dung (22.2%), and painting with mud (15.4%) (Fig. 2). The main reason for the modification was decoration (46.6%), followed by holiday celebration (28.6%), and dislike of insecticide smell (10%) (Fig. 3).

### Factors associated with house-wall modification after indoor residual spraying

Age, household head’s educational status, household head’s monthly income, household head’s level of knowledge about indoor residual spraying, information provided within two weeks before spraying, supervision after spraying, family history of malaria, previous experience of indoor residual spraying, other malaria prevention practices, stagnant water near house, and type of house roof showed association with house-wall modification after indoor residual spraying in the bivariate analysis at p-value < 0.25. The household head’s educational status, household head’s monthly income, household head’s level of knowledge about indoor residual spraying, information provided within two weeks before spraying, supervision after spraying, stagnant water found near the house, and type of house roof remained significant in the multivariable logistic regression model (Table 4).

**Table 4:** Factors associated with house-wall modification after indoor residual spraying in Shashogo district, southern Ethiopia, 2022

Variables	Wall modified		Crude OR (95% CI)	Adjusted OR (95% CI)
	No	Yes		
Household head’s educational status				
Can at least read and write	205	71	1	1
Cannot read and write	227	118	1.50 (1.06, 2.13)	1.76 (1.16, 2.68)
Household head’s monthly income (in Birr)				
≤ 1000	131	33	1	1
1001–2000	134	55	1.03 (0.63, 1.68)	0.92 (0.52, 1.66)
2001–3000	87	52	1.49 (0.93, 2.39)	1.66 (0.95, 2.89)
> 3000	80	49	2.43 (1.44, 4.09)	3.27 (1.78, 6.01)
Household head’s level of knowledge about residual indoor spraying				
High	240	35	1	1
Low	192	154	5.50 (3.64, 8.31)	3.81 (2.39, 6.06)
Information provided within two weeks before spraying				
Yes	303	85	1	1
No	129	104	2.87 (2.02, 4.09)	2.23 (1.44, 3.46)
Supervision after spraying				
Yes	295	67	1	1
No	137	122	3.92 (2.73, 5.62)	1.79 (1.14, 2.81)
Stagnant water found near house				
Yes	325	92	1	1
No	107	97	3.20 (2.24, 4.59)	3.36 (2.13, 5.30)
Type of house roof				
Corrugated iron sheet	136	111	1	1
Thatched	296	78	3.10 (2.17, 4.41)	1.82 (1.04, 3.18)

## Discussion

This study aimed to assess the prevalence of house-wall modification after indoor residual spraying before the end of residual lifespan and associated factors in Shashogo district, southern Ethiopia, 2022. The study has revealed that the prevalence of house-wall modification was significantly prevalent as compared with other similar studies. Moreover, the household head’s educational status, monthly income, low level of knowledge about indoor residual spraying, lack of information within two weeks before spraying, absence of supervision after spraying, absence of stagnant water near the house, and thatched roof were found to be significantly associated with house-wall modification after indoor residual spraying.

The prevalence of house-wall modification after residual spraying in the study area was found to be 30.4%. This was lower than a report from Tonga, South Africa (40%). The difference could be explained by socio-demographic variability; for example, the people of Tonga decorate their dwellings during December in preparation for Christmas festivities [8]. However, it was higher than findings from Africa (2.1–16.4%), Swaziland (10%), and other parts of Ethiopia (7.4–21%) [9–12]. Therefore, there is a need to strengthen the existing malaria control and prevention activities, like providing community education about indoor residual spraying, giving information before residual spraying, conducting supervision after residual spraying, and improving housing conditions. Besides, the impacts of cultural events should not be ignored.

This study has identified that there was a significant association between the household head’s educational status and house-wall modification after indoor residual spraying before the end of the residual lifespan. The odds of house-wall modification among households headed by those who could not read and write was about 1.79 times more likely compared to those who could at least read and write. This was supported by a study from Lume district, Oromia region which revealed that the respondents who could not read and write were 1.62 times more likely to modify indoor residual spraying intervention than those who could at least read and write [12]. Another study from Uganda has also revealed that the respondents who completed at least secondary education were about five times better at using indoor residual spraying than those who completed primary education [13]. This underscores that the improved educational history of a given community will be important to increase the uptake of malaria prevention efforts, including the use of indoor residual spraying.

Households with the highest socio-economic status may practice maintenance, paint, and decorate their houses compared to those households with the lowest socio-economic status. This study has revealed that the odds of house-wall modification after indoor residual spraying among households headed by those who earned a monthly income of more than birr 3000 were about three times more likely compared to those who earned a monthly income of a maximum of birr 1,000. However, the application of indoor residual spraying would play a great role in communities with poor socio-economic status. The poor are at risk of having a high incidence of malaria because of less access to preventive measures and health care, poor housing conditions that increase the entry of mosquitoes, and high susceptibility due to poor health and diet [14].

In this study, the odds of house-wall modification among households headed by those who had a low level of knowledge about indoor residual spraying were about four times more likely compared to those who had a high level of knowledge. This was consistent with a study from the Sidama zone, which revealed that the respondents who had a high level of knowledge about malaria were about nine times more likely to demonstrate good practices of malaria prevention and control compared to those who had a low level of knowledge [15]. Another study from Kabale district, Uganda, also showed that respondents with adequate indoor residual spraying knowledge were more likely to perform the desired practice compared to those with inadequate knowledge [16]. Furthermore, getting information within two weeks before spraying was also associated with house-wall modification in this study. The odds of house-wall modification among respondents who did not get information within two weeks before spraying were about two times more likely compared to those who got the information. It is well documented that improved knowledge and information would have an important impact on the prevention of poor practices against indoor residual spraying.

Regular supervision of malaria preventive activities has been known to promote good behaviours in communities and increase the effectiveness of interventions. In this study, the odds of house-wall modification among households that had not been supervised after indoor residual spraying were about two times more likely compared to those that had been supervised. This was in line with a finding from the Lume district, Oromia region, where those households that had not been supervised after indoor residual spraying were about 70% more likely to have a modified house-wall than those that had been supervised [12]. This indicated that providing supportive supervision to sprayed households might possibly decrease house-wall modification before the end of the potency period of the chemical, usually six months.

This study has also identified environmental factors, like distance from stagnant water sources and type of house roof, which would likely affect the status of the house-wall after indoor residual spraying. The odds of house-wall modification among households that were not found near stagnant water sources were about three times more likely compared to those that were located near stagnant water sources. This was supported by a study done in Wakiso district, Uganda, which revealed that participants who lived around vessels in a compound, near stagnant water, and in ponds were more likely to maintain sprayed insecticide than those who did not [17]. The possible explanation could be that the risk of malaria transmission would be higher as the mosquito breeding sites were located closer to households. Additionally, the odds of house-wall modification among thatched roof households were about two times more likely compared to those corrugated iron-sheet roof households. A comparable finding was observed in Tororo district, Uganda [18].

The findings of this study should be interpreted by considering its limitations. Recall biases on the date of supervision, the information provided before spraying, and the exact day of modification might be introduced. Social desirability bias might also affect the validity of the findings, in that households might report more socially acceptable responses. Moreover, the limitations that come with a cross-sectional study design need to be taken into consideration. Longitudinal follow-up studies are recommended in order to understand the time when house walls are being modified after the indoor residual spraying.

## Conclusion

This study has revealed that the prevalence of house-wall modification after indoor residual spraying before the end of the residual lifespan in the study area was higher compared to other studies in developing countries. Therefore, special emphasis should be given to providing community education about indoor residual spraying, conducting regular supervision before and after residual spraying, enforcing some punishment for modifying the house-wall before six months after spraying, and improving environmental and housing conditions.

## Data Availability

All relevant data are within the manuscript.
